# Multispacer Sequence Typing for *Mycobacterium bovis* Genotyping

**DOI:** 10.3389/fvets.2021.666283

**Published:** 2021-04-26

**Authors:** Érica Bravo Sales, Antônio Augusto Fonseca, Cristina Magalhães Gonçalves, Andrey Pereira Lage, Giovanna Ivo Andrade, Philip Noel Suffys, Harrison Magdinier Gomes, Natanael Lamas Dias, José Soares Ferreira Neto, Ana Marcia de Sá Guimarães, Marcos Bryan Heinemann

**Affiliations:** ^1^Laboratório Federal de Defesa Agropecuária de Minas Gerais, Pedro Leopoldo, Minas Gerais, Brazil; ^2^Escola de Veterinária, Universidade Federal de Minas Gerais, Belo Horizonte, Minas Gerais, Brazil; ^3^FioCruz, Rio de Janeiro, Rio de Janeiro, Brazil; ^4^Universidade de São Paulo, São Paulo, São Paulo, Brazil

**Keywords:** *Mycobactereium bovis*, bovine tuberculosis, genotype, epidemiology, MST

## Abstract

The molecular typing of *Mycobacterium bovis*, which causes bovine tuberculosis, can be accomplished by combining different polymorphic markers, contributing to its epidemiological investigation. Multispacer sequence typing (MST) is a sequencing-based method that employs intergenic regions susceptible to higher mutation rates given the low selection pressure. It has been applied to *M. tuberculosis*, but not to *M. bovis*. The aim of this study was to evaluate a MST for *M. bovis*. A total of 58 strains isolated from tissues with lesions suggestive of bovine tuberculosis, coming from cattle herds in six Brazilian states and four standard samples of *M. bovis* were typified employing the MST technique. Fourteen intergenic regions were used, and four types of genetic events were reported: single nucleotide mutation (SNP), insertion, deletion, and tandem repeat (TR). Seven loci were chosen for typing. Twenty-eight type sequences (ST) were identified, indicating type sequences (ST) were identified, indicating a 92.9% HGDI (Hunter Gaston Discriminatory Index). The data were used to analyze the evolutionary patterns of these isolates and correlate them to phylogeographic lineages based on the formation of clonal complexes generated from eBURST software. Later, we associated the MST with spoligotyping technique, currently considered the gold standard for classification of *M. bovis*. The results support the MST as an alternative method for genotyping of *M. bovis*. The method has the advantage of sequencing and the availability of sequences analyzed in public databases, which can be used by professionals around the world as a tool for further analysis. This was the first study to identify the variability of isolates of *M. bovis* by the MST method.

## Introduction

*Mycobacterium bovis* is the main pathogen of bovine tuberculosis (TB), an important disease of domestic cattle, which can also be associated with an underestimated number of human TB cases (zoonotic TB) and serious threats to wildlife conservation (1–4). Bovine TB is a disease of high health and economic relevance for livestock and is part of the group of notifiable diseases listed by the World Organization for Animal Health (OIE) (5). Infection routes in the cattle are influenced by factors such as age, environment, and hygiene practices, as the bacterium can be eliminated via various routes, such as respiratory droplets and aerosol, milk, feces, urine, vaginal secretions and/or semen (6). The presence of wildlife reservoirs in certain regions also hampers the control of the disease in livestock and translate into serious consequences for the preservation of wildlife species in multi-host systems (7). The disease directly affects livestock productivity, influencing international trade of animal products and, due to its zoonotic potential, is also of public health concern (8, 9).

*Mycobacterium bovis* belongs to the *Mycobacterium tuberculosis* complex (MTBC), a clonal group of mycobacterium species, lineages and/or ecotypes that cause similar diseases in a variety of mammalian hosts (10–12). Genomes of MTBC members are highly similar, with >99.9% identity over homologous regions, including the 16S rRNA gene, and absent remodeling through horizontal gene transfer or DNA recombination (10, 13, 14). This bacterial complex evolves only through SNPs (single nucleotide polymorphisms), short indels, large deletions, transposition of insertion sequence (IS) elements, and duplication of few paralogous gene families (10, 15, 16). Despite this high genetic similarity, species and lineages of the MTBC present variable phenotypes of host tropism and virulence (10–14).

Over the years, molecular genotyping techniques have significantly contributed to epidemiological studies of bovine TB, facilitating the knowledge of the spatial and temporal distribution of *M. bovis* strains, from populational structure to outbreak investigations (17, 18). Given the clonal nature of the MTBC, these techniques are based on few highly polymorphic regions of their genomes, including tandem repeats, a CRISPR locus, PGRS genes, and IS elements (17–21). The IS*6110*-based RFLP is a technique commonly used in *M. tuberculosis* genotyping, but of little application for *M. bovis* strains, as they carry only few IS*6110* copies in their genomes (22). Among the remaining techniques, spacer oligonucleotide typing (spoligotyping; based on the CRISPR locus) and variable number tandem repeat- mycobacterial interspersed repetitive units (MIRU-VNTR) have been the preferred combination for typing isolates of *M. bovis* in several countries (17–21). However, both techniques have limitations. Spoligotyping does not provide good resolution at the region or farm level, which limits its use for pathogen's transmission detection (17, 18). Spoligotyping is also sensitive to homoplasy, i.e., unrelated lineages presenting identical spoligotypes (23). This happens because the loss of a spacer region at the spoligotyping locus is a common event that can occur in strains that are not phylogenetically related (23, 24). While MIRU-VNTR has been reported to better discriminate among isolates at the farm level, there are 24 loci to choose from, and laboratories must frequently test which loci would better fit their reality for better discriminatory power (25). MIRU-VNTR is also influenced by homoplasy (24) and depending on the laboratory infrastructure, a 24 loci evaluation of *M. bovis* isolates to avoid homoplasy bias can be very laborious and time consuming (17).

Recently, *M. bovis* whole-genome sequencing (WGS) using next-generation sequencing platforms has been suggested to replace these genotyping assays (17, 18). However, WGS still faces many challenges related to its financial burden and requirement of specialized personnel, particularly in low and middle-income countries in which bovine TB is highly endemic. Most reported *M. bovis* WGS studies have been conducted in developed countries or in developing countries by researchers from high-income nations (26–32). Thus, albeit likely ideal, WGS use is still unreachable to many programs of bovine TB control and eradication around the world. Thus, there is a need for the continuous development of genotyping techniques with high resolution power. Accordingly, a multispacer sequence typing (MST) scheme for *M. tuberculosis* based on the Sanger sequencing of eight PCR-amplified intergenic regions, including four regions previously reported as VNTR regions, has been proposed (33). The aim of this study was to investigate the usefulness of this MST scheme to genotype 58 *M. bovis* isolates obtained from different Brazilian states.

## Materials and Methods

### Bacterial Isolates

A total of 58 cultured isolates of *M. bovis*, deposited in the sample bank of the National Agricultural Laboratory of Minas Gerais (Lanagro/MG), Brazil, were selected. These isolates were obtained from cattle herds located in six Brazilian states [Minas Gerais (*n* = 48), Goiás (*n* = 3), Mato Grosso-MT (*n* = 1), Rio Grande do Sul-RS (*n* = 1), Paraná-PR (*n* = 1) and São Paulo (*n* = 4)] between the years 2006 and 2010. First isolation of these *M. bovis* from clinical samples were performed in Lanagro/MG according to previous publications (34). Isolated strains were identified as *M. bovis* by using a PCR assay described by Sales et al. (35). Four additional laboratory strains [*M. bovis* CR01; *M. bovis* AN5 (36); *M. bovis* BCG CR13; *M. bovis* México CR36] were also selected. All procedures were performed in a Biosafety Level 3+ Laboratory (BSL-3+) located at the Farming National Laboratory (LANAGRO) Minas Gerais, Ministry of Agriculture, Livestock and Supply, Brazil. Tubes containing DNA were properly disinfected, removed from BSL3+ and stored at −20°C until further analysis.

### DNA Extraction and PCR Assays

A loop of each bacterial strain cultured in Stonebrink medium was subjected to a phenol-chloroform DNA extraction as previously described (37). Extracted DNA was subjected to conventional PCR assays of 14 intergenic spacer regions using previously described primer sequences (33). Each PCR assay was performed in a PX2 Thermal Cycler thermocycler (Milford, MA, USA). Briefly, 20 μL were prepared with the following concentrations of reagents: 10 pmol of each primer, 1.5 mM MgCl_2_, 10 mM dNTPs (Invitrogen, USA), 1 U Jump Start®Taq DNA Polymerase (Sigma, USA) and 1 X GoTaq Green® buffer (Promega, USA). DNA sample concentration added in each reaction was 100 ng/μL. As positive controls, we used DNA extracted from *M. bovis* strain AN5 (38), while negative controls consisted of ultrapure water. The conditions of the PCR assays determined for each pair of primers are shown in Supplementary Table 1. Amplification products were detected by gel visualization following electrophoresis.

### Sequencing and Data Analysis

The PCR products were purified using the PureLink® PCR Purification Kit (Invitrogen, USA) and sequenced using Big Dye® Terminator V.3.1 Cycle Sequencing Kit (Applied Biosystems, USA) according to the manufacturer's recommendations. Sequencing was performed in both directions in the 3130 Genetic Analyzer (Applied Biosystems, USA) equipment.

Sequence assemblies were performed with CAP3 program (39) using nucleotide data with quality higher than PHRED 20. Sequences were edited in Bioedit v.7.2.5 program (40). Subsequently, all sequences obtained for each primer pair were subjected to a multiple sequence alignment using Clustal Omega (https://www.ebi.ac.uk/Tools/msa/clustalo/) to verify the degree of identity between two or more sequences and the allelic diversity present in each spacer region. Each allele detected was assigned an identification number. For each individual DNA sample, the set of loci and their respective allele defined an allelic profile or sequence type (ST), which identified a clone. Bacterial isolates analyzed in this study were identified by numbers 1–58. The laboratory strains *M. bovis* CR01, AN5, CR13, CR36, and AF2122/97 (Genbank: NC002945.3) included in the analyses were represented by the numbers 59–63, respectively.

### Spoligotyping

The spoligotyping was performed as described by Kamerbeek (41) using spolygotyping kit by Ocimum Biosolutions. The identification of individual spoligotypes and the appointment of unpublished patterns were made using Mbovis.org website (www.mbovis.org) (42).

### Analysis of Genetic Relationship Among the Isolates

An evolutionary descent analysis of *M. bovis* isolates was performed using eBURST algorithm (43), available in Phyloviz software (44). Clonal complexes (CC) as defined by eBURST were identified and organized in dendrograms based on the comparison of the obtained allelic profiles of the tested *M. bovis* isolates. Briefly, CCs were determined by a simple model of clonal expansion and diversification based on the differences between these allelic profiles. Each CC was assigned a founder genotype that displays a more likely hypothetical pattern of evolutionary descent among the STs that make up this complex. The STs were organized into complexes by comparing the variation that exists among the loci of each founder genotype loci compared to other isolates (43). Accordingly, genotypic variations found in only one locus are called single locus variants (SLV), in two loci are called double locus variants (DLV), and in three loci are called triple locus variants (TLV) (43, 44). Confidence values for each SLV, DLV and TLV detected in each ST founder in a CC were assessed by bootstrapping (43). The geographic location and year of isolation of the strains were also included in the analysis.

### Phylogenetic Analysis

The phylogenetic reconstruction of *M. bovis* isolates was performed by PubMLST program (http://pubmlst.org/) using the Neighbor-Joining model. The program uses PHILIP package to create Neighbor-Joining and UPGMA trees based on allelic profiles determined in the study.

### Calculation of Allelic Diversity

The discrimination index (D) previously described by Hunter-Gaston (45) was used to calculate the allelic diversity at each locus and to assess genotypic diversity for each method employed (MST and spoligotyping).

## Results

### Multispacer Sequence Typing (MST)

**Figure 1:**
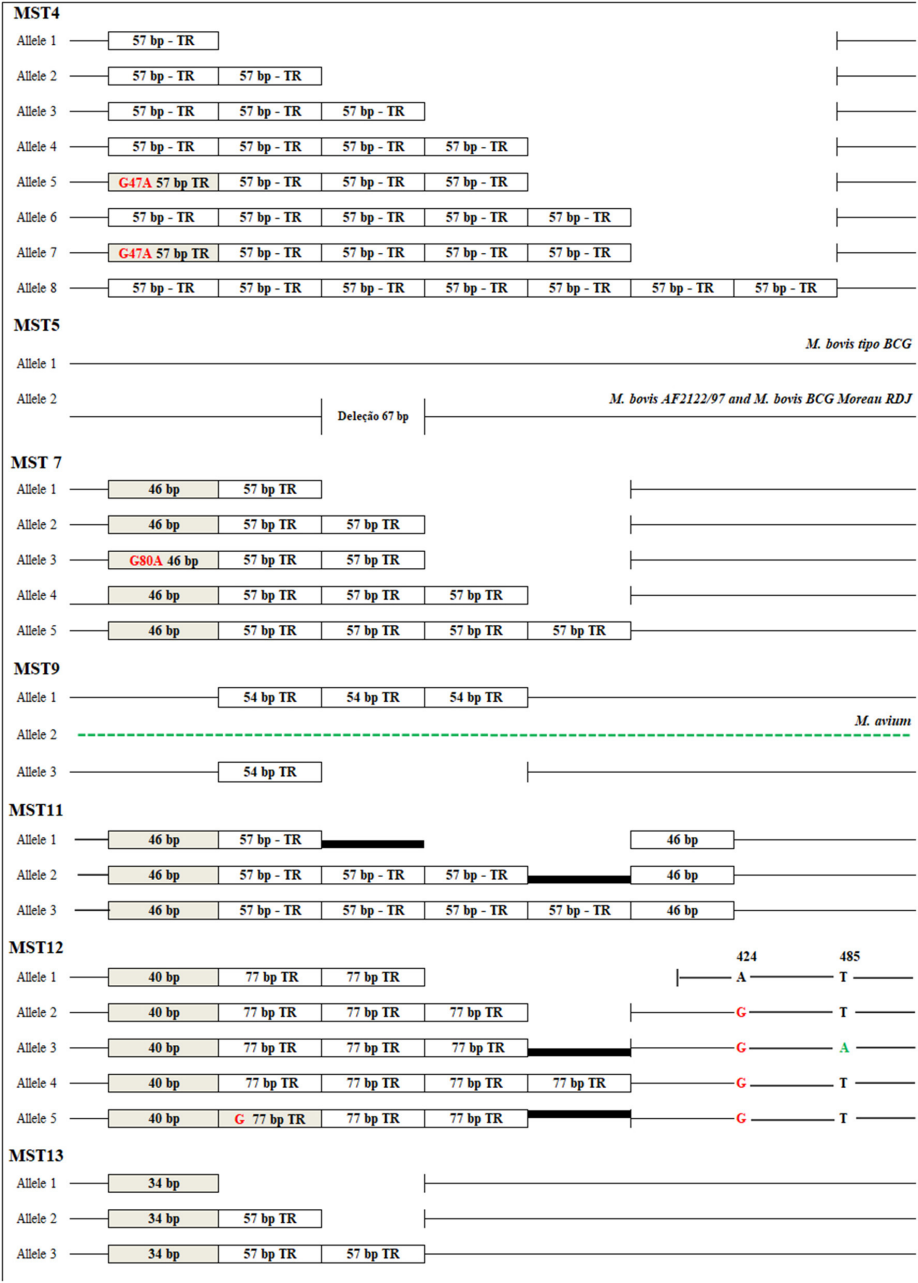
Alleles identified in seven intergenic spacers in *Mycobacterium bovis*. MST4 (ETR-B): eight alleles containing 1, 2, 3, 4, 5, and 7 copies of the 57 bp tandem repeat sequence (TR) and the presence of a SNP G47A (alleles 5 and 7). MST5: deletion of 67 bp. MST7: Five alleles containing from 2, 3, 4 and 5 copies of the 57 bp tandem repeat, a deletion of the first 11 bp repeat for all samples and a SNP G80A in allele 3. MST9: two alleles containing one and three repeat tandem copies of 54 bp, and a sequence represented by some polymorphisms in the genome of *M. bovis*, characterized by a different allele of a repeated region also present in the genome of *M. avium*. MST11: three alleles containing 3, 5, and 6 tandem repeats of 57 bp, the first and last repetition characterized by a deletion of 11 bp in all isolates. MST12: five alleles containing 3–5 repeat tandem copies of 77 bp with the first repetition characterized by a deletion of 37 bp in all isolates, the presence of a G76 base (allele 5), A424G SNP (allele 2–5), T485A SNP (allele 3) and a deletion of 18 bp after the last repeat (allele 1). MST13: three alleles containing from 1 to 3 copies of the 57 bp and a 23 bp deletion in the first repetition in all isolates.

Fourteen intergenic spacer regions were analyzed in the selected *M. bovis* isolates. Regions MST1, MST2, MST3, MST6, and MST8 showed no sequence variation in an initial evaluation of 15 randomly chosen *M. bovis* isolates of the collection and were then excluded from the study. In addition, after multiple attempts, the PCR assay of the MST10 region failed to amplify appropriate PCR products, and this region was also excluded from the study. For the MST14 region, Sanger sequencing was not able to cover the whole extent of the region, hampering the identification of possible polymorphic positions for some of the samples and was also excluded from further evaluation. Finally, the remaining regions MST4, MST5, MST7, MST9, MST11, MST12, and MST13 proved quite variable, with a total of 28 mutational events, including single nucleotide polymorphisms (SNP), insertions, deletions, and tandem repeats. Figure 1 shows the alleles identified for each of the seven evaluated spacer regions.

### Definition of Genetic Profiles

Multiple sequence alignment of the DNA sequences of all *M. bovis* isolates for each locus allowed the identification of up to 8 different alleles which received individual identifier numbers (Figure 1). The grouping of all loci for each individual defined an allelic profile that was represented by a sequence type (ST). For the 63 isolates of *M. bovis* studied, 28 STs were found, where eight were represented by more than one *M. bovis* isolate, and 20 were described as orphan STs, as shown in Table 1.

**Table 1:** Twenty-eight (28) ST identified by the comparison of genotypic profiles of 63 isolates of *M. bovis*.

**ST**	**Number of *M. bovis* isolates**	**Allelic profile**							**%**
		**MST4**	**MST5**	**MST7**	**MST9**	**MST11**	**MST12**	**MST13**	
6	12	4	2	4	1	2	2	3	19.05
17	9	4	2	4	1	1	2	3	14.29
1	5	3	2	4	1	2	2	3	7.94
10	5	4	2	4	1	1	2	2	7.94
19	4	4	2	4	1	1	5	3	6.35
11	4	6	2	4	1	2	2	3	6.35
14	2	4	2	4	1	1	3	3	3.17
25	2	4	2	4	1	2	5	3	3.17
45	1	1	2	4	1	1	2	3	1.59
30	1	2	2	4	1	1	2	2	1.59
61	1	3	2	1	1	2	2	1	1.59
54	1	3	2	4	1	1	2	3	1.59
31	1	4	1	4	1	2	2	1	1.59
5	1	4	2	2	2	2	2	3	1.59
39	1	4	2	4	1	1	2	1	1.59
9	1	4	2	4	1	3	4	3	1.59
4	1	4	2	4	2	1	2	3	1.59
13	1	5	1	4	1	1	2	2	1.59
12	1	5	2	4	1	2	2	3	1.59
52	1	6	1	4	1	2	2	3	1.59
63	1	6	1	4	1	2	4	3	1.59
29	1	6	2	4	1	1	2	2	1.59
62	1	6	2	4	1	2	1	2	1.59
59	1	6	2	4	1	2	2	2	1.59
60	1	6	2	4	1	2	4	2	1.59
58	1	7	2	3	3	2	2	3	1.59
55	1	7	2	4	1	2	2	3	1.59
34	1	8	2	4	1	2	2	1	1.59

*ST identifies the sequence type featuring a genotypic profile (or allelic)*.

^*^Displays standard strains of M. bovis without source information. % Percentage of M. bovis isolates with corresponding ST.

### Evolutionary Descent Analysis

**Figure 2:**
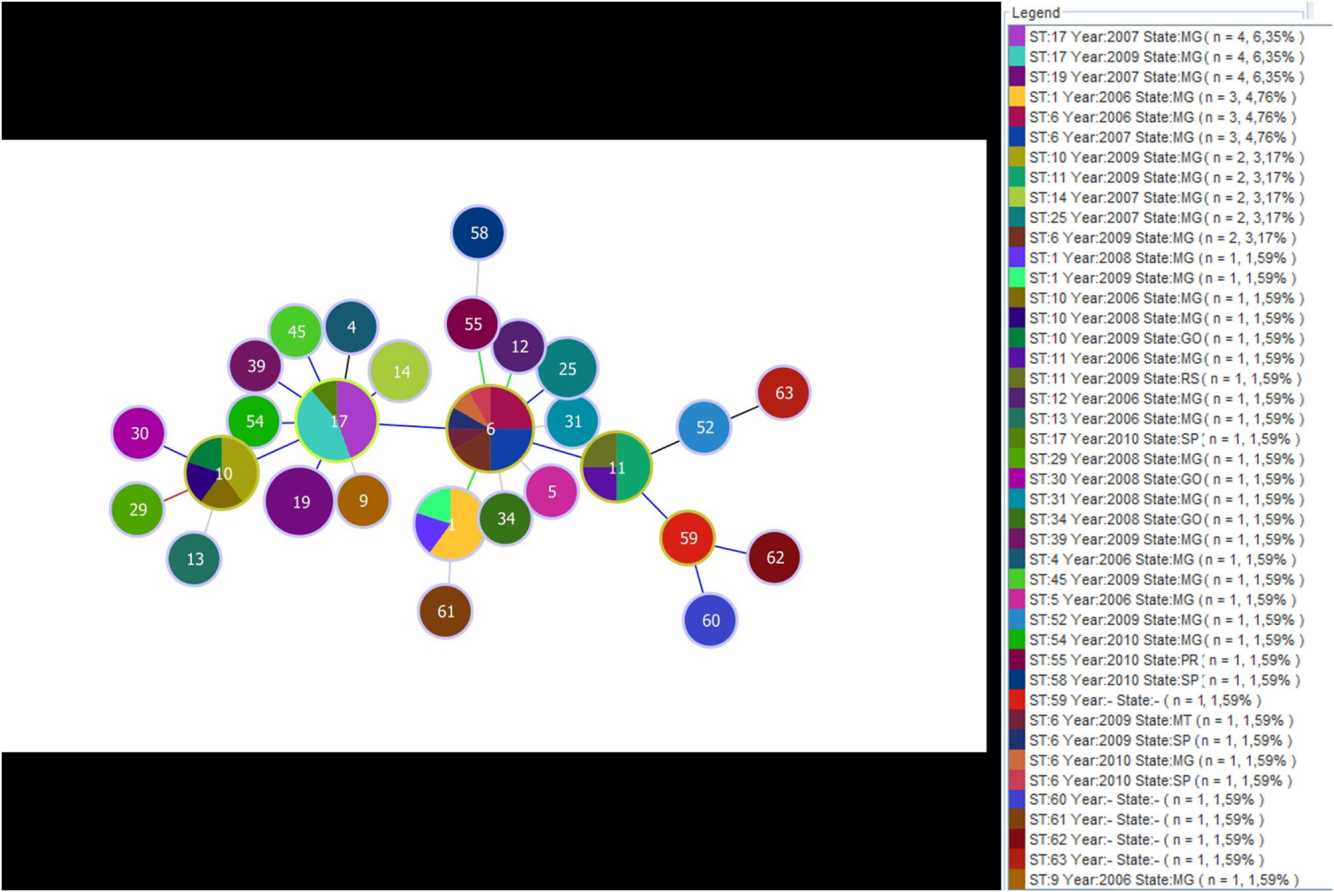
Minimum spanning tree exhibiting the formation of clonal complexes according to genotypic profiles obtained by the multi sequence typing of *Mycobacterium bovis*. Distribution of 58 samples of *M. bovis* isolated from the years 2006 to 2010, four standard samples of *M. bovis* (CR01, CR02, CR13, and CR36) and the strain *M. bovis* (AF2122/97). The dendrogram reveals the formation of five clonal complexes ST6, ST10, ST11, ST17, and ST59 that formed from the comparison of 28 ST. In the subtitle “N” figure indicates the number of isolates with the same genotypic profile, as well as the size of the circles (the larger the circle, the larger the population represented). Circles with more than one color profile indicate the same genotype isolated from different regions and year of isolation of the pathogen.

The STs detected in the study were used to determine the genetic relationship of *M. bovis* isolates. A total of five CCs (ST6, ST10, ST11, ST17, and ST59) were detected (Figure 2). ST17, consisting of nine *M. bovis* isolates, being eight from Minas Gerais state and one from São Paulo state, was appointed as the founder CC of the whole group, as it had the highest number of SLVs (ST4, ST6, ST10, ST14, ST19, ST39, ST45, and ST54, including 24 *M. bovis* isolates from Minas Gerais, two from São Paulo and one from Mato Grosso) with diverging genotypes in only one of seven loci. ST6 was appointed the largest CC and presented the highest frequency of isolates with the same genotypic profile from different geographical locations: 10 *M. bovis* isolates from Minas Gerais, two from São Paulo, and one from Mato Grosso. This complex presented six SLVs, which expressed close relationship to ST1, ST11, ST12, ST17, ST25, and ST55, with 22 *M. bovis* isolates from the states of Minas Gerais (*n* = 19), São Paulo (*n* = 1), Goiás (*n* = 1), and Paraná (*n* = 1). ST11, consisting of four strains, coming from Minas Gerais and Goiás, is one of the SLVs of ST6 that has diversified producing their own SLV, characterized by members with unique genotypic profiles. Interestingly, the single *M. bovis* isolate from Rio Grande do Sul, the only state not bordering any of the states evaluated herein, having unique genotype (ST13) without associated SLVs, DLVs or TLVs.

### Spoligotyping and MST

Spoligotyping was performed for 55 of the 63 *M. bovis* isolates that composed the study. A total of 22 spoligotypes were detected, 19 of which were already mentioned in the literature. A total of eight clusters (G1–G8) were formed, representing 74.54% (41/55) of the *M. bovis* isolates. The remaining 14 (24.45%) *M. bovis* isolates had the spoligotype described as orphans (Table 2). SB2211, SB2212, and SB2213, found in samples from Minas Gerais, São Paulo, and Mato Grosso, respectively, have never been described prior to this study. SB1135 was the most frequent spoligotype (n = 12), followed by SB0121 (*n* = 9), SB0295 (*n* = 6), and SB0881 (*n* = 5). SB1135 had samples only from Minas Gerais, while SB0121 and SB0295 were represented by Minas Gerais and São Paulo; and SB0881 by Minas Gerais and Goiás.

**Table 2:** Spoligotypes of 55 *Mycobacterium bovis* isolates.

	**Spoligotypes**	**# *M. bovis*** **isolates**	**Clustering**	**Orphan** **profiles**	**Patterns description**	**State**
1	SB0120	2	G1	–	■■□■■■■■□■■■■■■□■■■■■■■■■■■■■■■■■■■■■■□□□□□	GO/^*^
2	SB0121	9	G2	–	■■□■■■■■□■■■■■■□■■■■□■■■■■■■■■■■■■■■■■□□□□□	MG/SP
3	SB0295	6	G3	–	■■□■■■■■□■■■■■■□■■■■□■■■■■■■■■■■■■■■□■□□□□□	MG/SP
4	SB0333	2	G4	–	■■□■■□■■□■■■■□□□■■■■□■■■■■■■■■■■■■■■■■□□□□□	MG
5	SB0881	5	G5	–	■■□■■■■■□■■■■■■□■■■■□■■■■□□□□□□■■■■■■■□□□□□	MG/GO
6	SB1033	3	G6	–	■■□■■□■□□□□□■■■□■■■■■■■■■■■■■■■■■□■■■■□□□□□	MG
7	SB1135	12	G7	–	■■□■■■■□□■■■■■■□■■■■□■■■■■■■■■■■■■■■□■□□□□□	MG
8	SB1139	2	G8	–	■■□■■■■■□■■■■■■□■■■■■■■■□□□□□□□□□□■■■■□□□□□	MG/SP
9	SB0140	1		9	■■□■■□■□□□□□■■■□■■■■■■■■■■■■■■■■■■■■■■□□□□□	MG
10	SB0267	1		10	■■□■□■■■□■□■■■■□■■□■■■■■■■■■■■■■■■■■■■□□□□□	MG
11	SB0268	1		11	■■□■□■■■□■□□■■■□■■■■■□■■■■■■■■■■■■■■■■□□□□□	^^*^^*^^
12	SB0486	1		12	■■□■■□■□□□□□□□■□■■■■■■■■■■■■■■■■■■■■■■□□□□□	RS
13	SB0849	1		13	■■□■□■■■□■■■■■■□■■■■■■■■■■■■■■■■■■■■■■□□□□□	MG
14	SB1050	1		14	■■□■■■■■□■■■■■■□■■■■□■■■■■■■■□□□□□■■■■□□□□□	MG
15	SB1093	1		15	■■□□■■■■□■■■■■■□■■■■□■■■■■■■■■■■■■■■■■□□□□□	MG
16	SB1136	1		16	□□□□□□□□□□□■■■■□■■■■■■■■■■■■■■■■■■■■■■□□□□□	MG
17	SB1145	1		17	■■□□□□□■□■■■■■■□■■■■□■■■■□□□□■■■■■■■■■□□□□□	GO
18	SB1369	1		18	■■□■■■■■□■■■■■■□■■■■□■■■■■■■■■□□□□■■■■□□□□□	PR
19	SB1802	1		19	■■□■□■■■□■□■■■■□■□■■■■■■■■■■■■■■■■■■■■□□□□□	MG
20	SB2211	1		20	■■□■■■■■□■■■■■■□■■■■□■■■■■■■□□■■■■■■■■□□□□□	SP
21	SB2212	1		21	■■□■■■■■□□■■■■■□■■■■□■■■■■■■■□□□□□■■■■□□□□□	MG
22	SB2213	1		22	■■□□■■■■□□■■■■■□■■■■□■■■■■■■■■■■■■■■■■□□□□□	MT

* *Laboratory strain M. bovis BCG CR13*

** *Laboratory strain M. bovis Mexico CR36. GO, Goiás; MG, Minas Gerais; RS, Rio Grande do Sul; SP, São Paulo; MT, Mato Grosso*.

**Figure 3:**
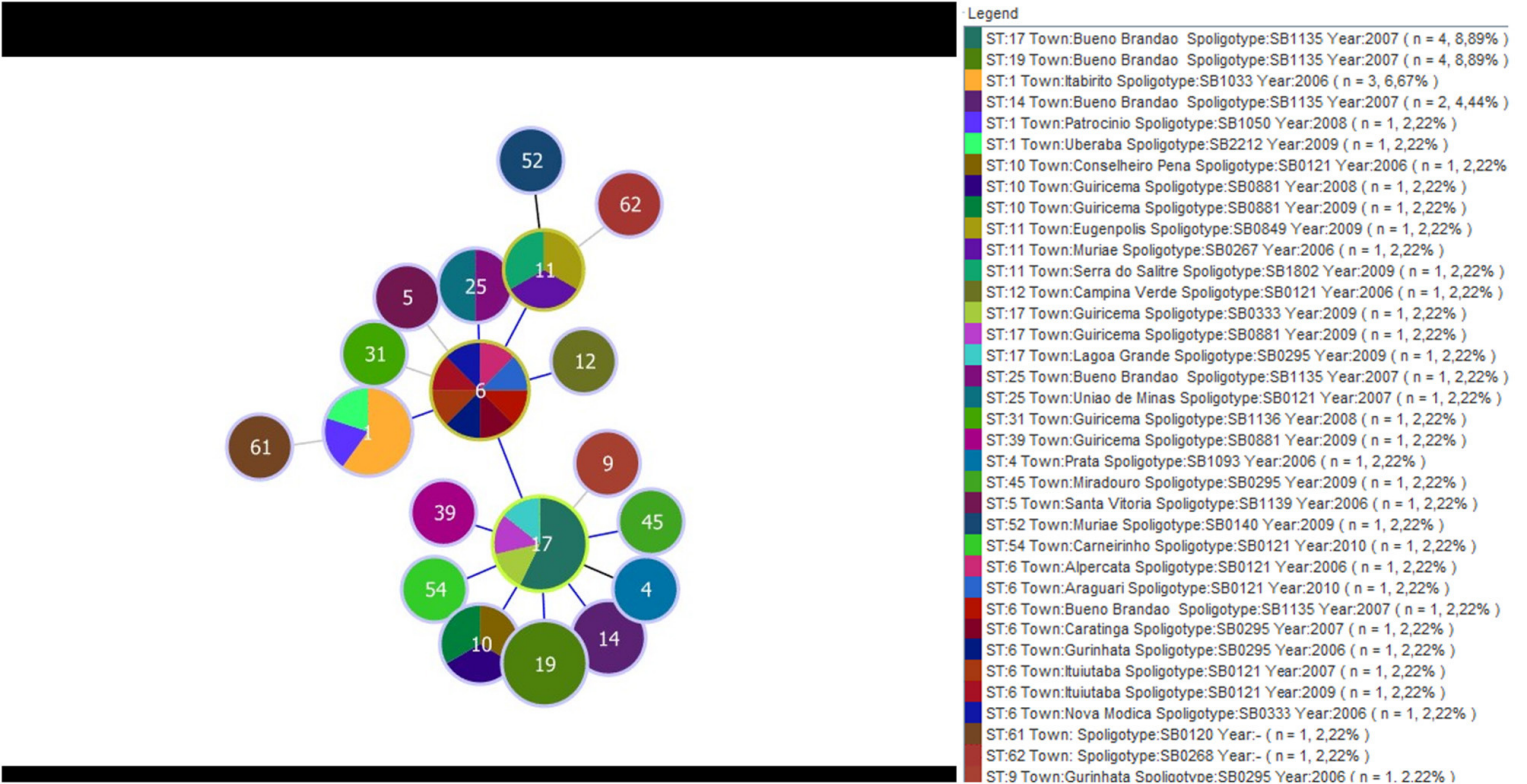
Minimum spanning tree displaying the distribution of 19 type sequences and 17 spoligotypes determined by the MST and Spoligotyping, respectively. The data were obtained from 43 isolates of *M. bovis* from 22 municipalities in MG and isolates between the years 2006 and 2010. The colors represent the different spolitypes and ‘*n*' indicates the number of isolates with the same genotype profile.

A separate analysis contrasting MST and Spoligotyping was also performed using samples from Minas Gerais only, which corresponded to 43 of the 58 *M. bovis*. These samples came from 22 different towns and were isolated between 2006 and 2010. A total of 19 STs and 17 spoligotypes were identified. The eBURST displayed ST6 as the main clonal complex, showing for this CC three SLVs (ST17, ST11, and ST1). ST6 reported eight samples from seven different cities, and four spoligotypes (SB0295, SB0121, SB1135, and SB0333); ST17 reported seven samples from three different cities, and four spoligotypes (SB1135, SB0295, SB0333, and SB0881); ST1 composed five *M. bovis* isolates, from three cities, and three spoligotypes (SB1033, SB1050, and SB2212); and ST11, which depicted three samples, each one from a different city and showed orphans spoligotypes (SB0267, SB0849, and SB1802). The three most frequent spoligotypes were SB1135, represented by 12 samples isolated in 2007, all of them from a same town; SB0121 which was reported in eight samples, from seven cities and isolated in 2006, 2007, 2009, and 2010; and SB0295 reported in five samples, from four cities and isolated in 2008 and 2009 (Figure 3).

### Phylogenetic Analysis

**Figure 4:**
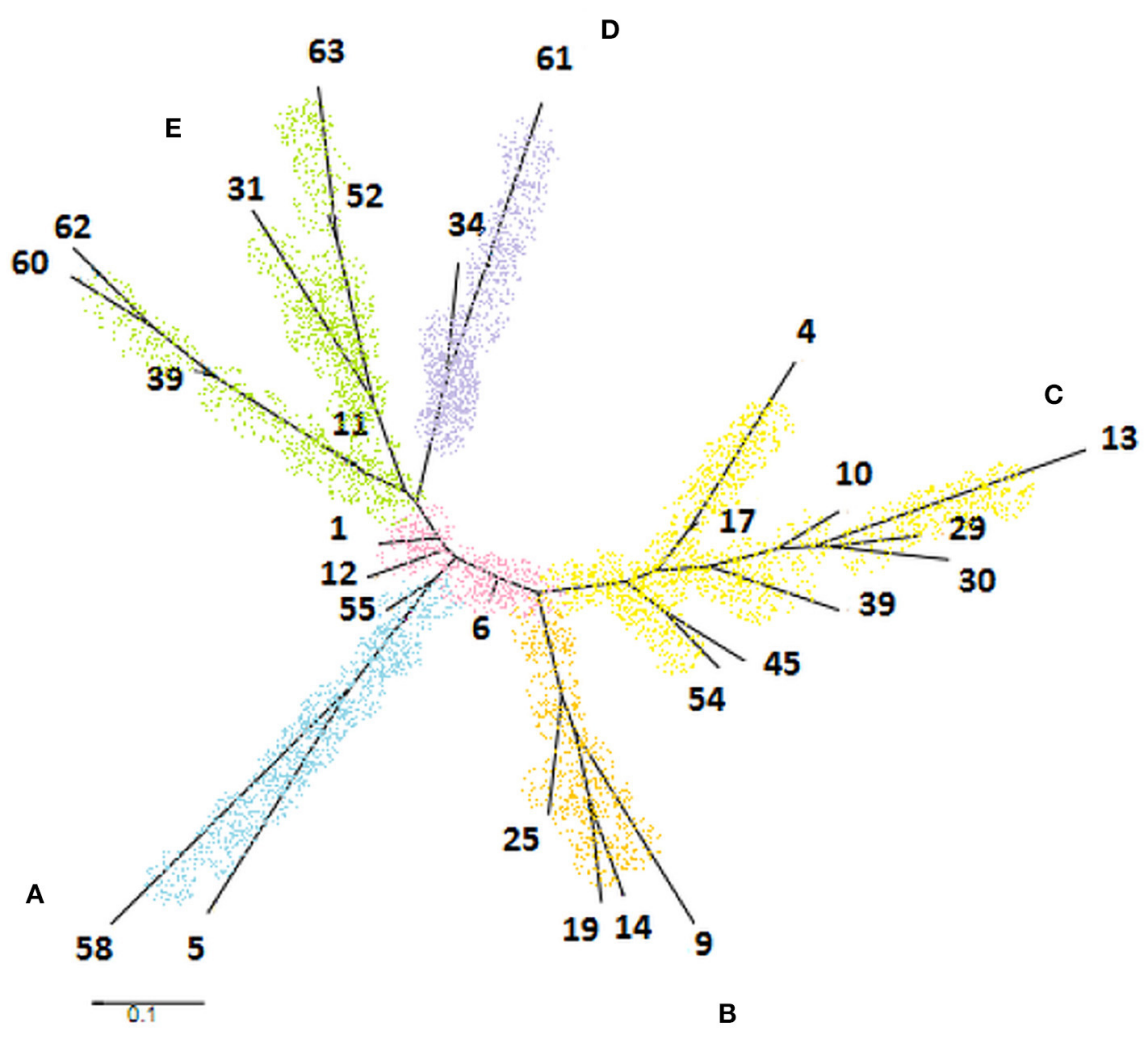
Phylogenetic tree generated by PubMLST program based on 28 sequence types of *Mycobacterium bovis* obtained in the study. It was observed the formation of five clades identified by letters and colors as follows: clade **A** (blue), clade **B** (orange), clade **C** (yellow), clade **D** (purple), and clade **E** (green).

The phylogenetic tree was generated from 28 STs constituting a cladogram where each ST was represented by its number (Figure 4). Overall, *M. bovis* isolates were similarly clustered in the same groups formed by eBURST. It was observed that ST1, ST6, and ST12 were closely located in the center of the tree. When comparing this phylogenetic tree with the genotypic profiles of each ST, the genetic variation was limited by the difference in a single locus, admitting the close relationship between these isolates, which coincide with data observed previously by eBURST. The formation of five clades distributed in the tree edges was observed and the relationship between these clades was generated by variation of three or more loci identified by MST. Clade A was represented by the sequence type coming from Minas Gerais, São Paulo, and Paraná. Clade B was formed only by isolates from Minas Gerais. Clade C was represented by members of Minas Gerais and Goiás. And finally, clade D was represented by a single sample from Goiás and *M. bovis* BCG CR13, while clade E was formed by laboratory strains *M. bovis* CR01, CR02, AN5, CR36, and AF2122/97.

The clade A, marked by the ST5 and ST58, showed the same pattern of spoligotype (SB1139), so as clade D, composed of ST34 and ST61 of spoligotype SB0120. The clade B, formed by ST9, ST14, ST19, and ST25, is composed of spoligotypes SB0295 and SB1135. These two spoligotypes differ only by the absence of the spacer eight in SB1135 compared to SB0295. Clade C includes eight different ST and four spoligotypes. The three ST located in the base of the tree were well-diversified in terms of spoligotypes patterns, corresponding to the high diversity of the ST6. Interestingly, although highly diverse in terms of ST types, these isolates differ only by SLVs. Clade E presented high diversity both of spoligotypes and of genotypic profiles described for ST (Figure 4).

### Calculation Hunter and Gaston

The discriminatory power of MST and spoligotyping techniques were calculated according to the Hunter-Gaston discrimination index (45). The determination was based on the number of genotypic profiles defined during the test and the relative frequencies of each profile. For MST, the calculations were made for the 63 strains of *M. bovis* and 28 ST resulting in a 92.9% HGDI. Yet for spoligotype, reducing to 55 the number of isolates and to 22 the number of patterns, the index lowered to 91%.

## Discussion

The 14 spacer regions analyzed in this study for isolates of *M. bovis* were first described by Djelouadji et al. (33) to characterize *M. tuberculosis* isolates. The decision of analyzing them was taken based on the genetic similarity corresponding to 99.95% between these two species. The markers used are from intergenic regions susceptible to higher mutation rates given the low selection pressure. As the bovine bacillus has a broad host range compared to human germ and evolution occur under various conditions for both, we studied the 14 regions to find the most variable in *M. bovis*.

Genotyping by the MST included the evaluation of 14 intergenic spacer regions, 58 representative samples of *M. bovis*, four standard samples and AF2122/97 strain obtained from GenBank. A total of seven spacers showed genetic variability for the analyzed isolates. Four of them were previously described as Exact Tandem Repeat (ETR) defining ETR-B (MST4), ETR-C (MST11), ETR-D also called MIRU4 (MST12) (46, 47) and Mtub21 (MST13) (48). The other three spacers used were MST5, MST7, and MST9. The results showed the occurrence of 28 genetic events, distributed among deletions, insertions, variation in the number of tandem repeats and point mutations. Sequencing shows the exact number of tandem repeats present in each sequence, and also determines stable markers within them such as SNPs (49) considered very important for studies of genetic events such as phylogeny.

Drancourt et al. (50) used ETR D-region (MST12) to differentiate eight *M. tuberculosis* complex members by sequencing. The region MST12 (ERT-D) in this study revealed the presence of five alleles characterized by a 40 bp sequence in all isolates, followed by 2–4 copies of the tandem repeat of 77 bp. It was also revealed G76 insertion of a base, an SNP A424G, an SNP T485A and a deletion of 18 bp after the last repetition of the first allele. These events were detected by comparison with the genome of the strain AF2122/97 analyzed *in silico* (Figure 1). These data, when compared to sequences characterized by (50) available in Genbank (EU180228- EU180234), showed that profiles found for *M. bovis* in this study were different from the profiles described by the author for the number of repetitions and for SNP.

MST performed for isolates of *M. tuberculosis* defined eight of 14 loci analyzed as variables (MST1, MST2, MST3, MST4, MST8, MST11, MST12, and MST13). In our study, four of these (MST1, MST2, MST3, and MST8) were excluded from our study due to low variability in the isolates of *M. bovis*. Djelouadji et al. (33) identified thirteen alleles in these regions that constituted genetic events of deletions and nucleotide substitutions. The other four spacers (MST4, MST11, MST12, and MST13) were characterized mainly by tandem repeats and showed a different profile found for *M. bovis*. A frequently observed event was the presence of a point mutation at the first base of many of the repetitions, which did not occur in any of the isolates of *M. bovis* studied in this work. MST12 and MST13 had a larger number of alleles for *M. tuberculosis*, while MST4 and MST12 were more variable for *M. bovis*.

The low genetic variability observed in the excluded MST may be related to the sampling bias in samples from Minas Gerais in relation to other states. The existing allelic diversity among *M. bovis* and *M. tuberculosis* may be due to the wide range of hosts of the bacillus of bovine, evolving in different environments and under different conditions (51). The population size and genetic diversity found in *M. tuberculosis* strains in relation to *M. bovis* can also be representative of these differences.

The most common spoligotypes in our study were SB1135 (21.8%), SB0121 (16.36%), SB0295 (10.9%) and SB0881 (9%) (Table 2). Of these, SB1135 has been described so far only in Brazil (Mbovis.org). Except for SB1135, the other three SB were also reported by Parreiras et al. (52), as well as SB0120, SB1802, SB1145, SB1033, and SB0267 described as an orphan or less frequent. Corroborating our findings, Rodriguez et al. (53) described SB0295 and SB0121 as the majority SB in a study conducted on a national level, as well as (38) which also recorded the occurrence of SB0295 spoligotype in the country. There have been reports of both in many Brazilian isolates and other Latin American countries (54). SB0881 was described previously in Brazil (55), France (56) and Spain (57). Haddad et al. (56) described the SB0295 and SB0121 in Holland in Belgium. SB0120 and SB0295 were also reported among animal wildlife and domestic species, in southern Spain (58), while SB0121 was identified in cattle and wild fauna in the Iberian Peninsula (59) and had also occurred in Mexico and Venezuela (54).

On the less frequent spoligotypes or orphans, SB0140 was described by Smith et al. (60, 61) as one of the most frequent patterns found throughout the world, particularly in Europe and the British Isles; however, the pattern was observed only in a single isolate in our study. SB0140 was also the most frequent pattern according to a study by (54) using isolates from Latin America. SB1145 and SB1802 were only isolated in Brazil, previously reported in Minas Gerais, also with low frequency (52).

CCs were formed and organized from the association of seven genetic regions. The use of clonal complexes as a tool for population analysis is important because it allows us to recognize specific subpopulations or clones that have unique genetic characteristics. Epidemiologically related isolates are derived from the clonal expansion of a single cell, resulting in common characteristics (62). By analyzing MST in association with spoligotyping we observed that some isolates were closely related with exclusive MST profiles and spoligotypes while others, coming from the same region and year, showed different profiles and patterns. The results identified 15 orphans' profiles for MST, 14 orphan spoligotypes and eight groups represented by more than one isolate with equal genotypes in both techniques (Figure 2). Similar results were obtained by Parreiras et al. (52) by associating spoligotyping and MIRU-VNTR. The procedure used for this kind of occurrence typed by VNTR in association with spoligotyping is to rearrange the isolates identified by the same VNTR profiles groups geographically located at spoligotyping (60), thereby obtaining more data on the phylogeny of the bovine bacillus.

The importance of finding an alternative method for genotyping *M. bovis* is a result of the occurrence of remarkably diverse populations in different countries, making it difficult to determine a set of useful markers that meets all regions (59). Regional differences in the discriminatory power of genetic markers make it necessary to define an optimal combination of markers that can be used in a particular region or country (63). The different patterns obtained indicate the variety of strains that infect cattle from different geographical areas.

In this study, seven polymorphic loci MST were characterized as variables for *M. bovis*. The observed molecular variation was explored to determine the evolutionary relationships of this pathogen. The results showed that the tested regions can be a promising tool in epidemiological studies. IS*6110* RFLP techniques, spoligotyping, and VNTR have been used as a reference for studying the diversity of species of the *M. tuberculosis* complex (55). However, these methods do not recognize the genetic diversity of strains analyzed as sequencing-based methods capable of identifying all the genetic events present in the molecular marker. The MST method proposed offers the advantage of sequencing, which allows for the detection of different genetic events observed in one sequence. The typing methods performed based on sequencing provide important data on evolutionary forces that shape bacterial populations (64). Bing et al. (65) determined that a methodology based on sequencing of non-constotive genes (MLSA) can be used for molecular typing, in addition to being an excellent method for use in phylogenetic analyzes, for epidemiological and surveillance studies.

Our results demonstrate that the number of samples needs to be increased to provide additional data to monitor the formation and expansion of clonal complexes according to geographic location and year of isolation. This was the first study to identify the variability of isolates of *M. bovis* by the MST method. The results indicated that the method works to typify differentiate isolates of *M. bovis* using fewer than those used in VNTR or special reagents like the membranes used in spoligotyping.

## Conclusions

Genotyping by MST can contribute to epidemiological studies of *M. bovis*. The method was efficient to detect the genetic variability present in the sequences analyzed and to infer evolutionary relationships in the short term. The method was also suitable to correlate geographical location and phylogenetic data of *M. bovis* isolates used in this study.

## Data Availability Statement

The raw data supporting the conclusions of this article will be made available by the authors, without undue reservation.

## Author Contributions

ES, CG, GA, HG, ND, and PS performed the all-laboratory tests. ES, AF, and AL performed the interpretation of DNA sequencing results. AF, MH, and AL wrote the manuscript and AG did the translation. MH, AG, PS, and JF accurately reviewed the manuscript. All authors have read and approved the final version of the manuscript.

## Conflict of Interest

The authors declare that the research was conducted in the absence of any commercial or financial relationships that could be construed as a potential conflict of interest.
